# Childhood experience profiles and their impact on depression–burnout networks among nurses: a latent class and network analysis

**DOI:** 10.1186/s12912-025-03889-x

**Published:** 2025-09-29

**Authors:** Jiao-Mei Xue, Ping-Zhen Lin, Wei Guo, Li-Hui Yang

**Affiliations:** 1 School of Health and Elderly Care, Shandong Women’s University, Jinan, 250300 China; 2 Department of Nursing, Quanzhou First Hospital, Quanzhou, 362000 China; 3 School of Education, Shandong Women’s University, Jinan, 250300 China; 4 Rare Cancer Ward, Shandong Cancer Hospital and Institute, Jinan, 250117 China

**Keywords:** Depression, Burnout, Childhood experiences, Latent class analysis, Network analysis, Nurse

## Abstract

**Background:**

This study aimed to use a person-centered approach to differentiate types of childhood experiences among nurses and explore whether the relationship between depression and burnout differs across these groups.

**Methods:**

This study employed a cross-sectional design and conducted a questionnaire survey of 866 nurses using convenience sampling. Nurses in a general hospital were surveyed using the Adverse Childhood Experience (ACE) and Benevolent Childhood Experience (BCE) scales, Patient Health Questionnaire-9 (PHQ-9), and Maslach Burnout Inventory (MBI). Latent class analysis in Mplus 8.3 classified nurses by ACEs/BCEs patterns, and network analysis was conducted using the bootnet package in R 4.3.2 to visualize complex interactions between depression and burnout by constructing network diagrams.

**Results:**

Latent class analysis identified two childhood experience profiles: *Low ACEs/High BCEs* (*n* = 648) and *Moderate ACEs/Low BCEs* (*n* = 218). Network analysis revealed stronger overall connections between depression and burnout symptoms in the Moderate ACEs/Low BCEs group, with Emotional Exhaustion acted as a bridge symptom, transmitting distress between depression and burnout, and thus representing a critical target for intervention. while Cynicism was also a key bridging symptom specifically in the *Moderate ACEs/Low BCEs* group.

**Conclusions:**

These findings demonstrate that Chinese nurses’ childhood experiences cluster into distinct patterns, with Emotional Exhaustion and Cynicism representing critical intervention targets. Hospital administrators should prioritize monitoring emotional exhaustion and reducing cynicism, particularly among nurses with *moderate ACEs/low BCEs*, to safeguard workforce stability and patient care.

**Clinical trial number:**

Not applicable.

**Supplementary Information:**

The online version contains supplementary material available at 10.1186/s12912-025-03889-x.

## Background

Clinical nurses face significant psychological strain due to heavy workloads, complex relationships, and irregular shifts, leading to high rates of depression (28.4%-44.6%) and burnout (29.5%-60.99%) [1–5]. Notably, the latest edition of the International Classification of Diseases (ICD-11) by the World Health Organization classifies burnout as an occupational health syndrome [6].

The prevalence of comorbid depression and burnout is relatively high, and there is significant overlap in symptoms between the two conditions, particularly between fatigue in depression and emotional exhaustion in burnout [7, 8]. Furthermore, depression and burnout have been demonstrated to have a mutually reinforcing relationship [9]. The independent prediction of nurses’ QoL and patients’ QoL based on both depression and burnout is supported by the literature [10–12]. Therefore, it is important to explore the relationship between depression and burnout in nurses.

Traditional psychopathology theory views mental disorder symptoms as isolated, interchangeable units. Conversely, network theory posits symptoms are interconnected, interact, and collectively form a complex system, where the symptom network itself defines the disorder [13]. Symptoms can be activated by neighboring symptoms or external factors. Network analysis maps symptom relationships, identifies core symptoms, and detects bridging symptoms across disorders. This enables targeted interventions to prevent/alleviate psychological problems among nurses. Accordingly, this study employed a network analysis to elucidate the relationship between depression and burnout.

The relationship between depression and burnout is influenced by several factors. Among these factors, childhood experiences have significant environmental influences. Critically, childhood experiences shape mental health trajectories: Adverse Childhood Experiences (ACEs) increase depression/burnout risk [14, 15], while Benevolent Childhood Experiences (BCEs) independently promote psychological resilience [16, 17]. Notably, adverse and benevolent childhood experiences are not mutually exclusive. It is also possible that individuals who have experienced adverse childhood circumstances may have had positive experiences [18]. Most previous studies on childhood experiences have been variable-centered, preventing them from focusing on individual specificity. By contrast, latent class analysis (LCA) is an individual-centered approach that classifies individuals according to their distinct response patterns to observed indicators, thereby achieving the objective of identifying group heterogeneity. To date, only two studies have conducted latent class analyses of ACEs and BCEs [19, 20]. Both studies focused on adult populations in Western countries and identified four latent classes. Three of these classes were consistent across both studies: (1) low ACEs/high BCEs, which was the largest proportion in both studies, (2) moderate ACEs/high BCEs, and (3) high ACEs/moderate BCEs, which was the smallest proportion in both studies. Johnson’s study identified a fourth category as moderate ACEs/low BCEs, and Cain’s study identified a fourth category as low ACEs/moderate BCEs. Johnson’s study found that adversity significantly impacts individual mental health and that positive experiences struggle to mitigate this effect. However, Cain’s study identified the protective role of BCEs.

Furthermore, the cultural context must be considered when interpreting findings related to emotional distress and childhood adversity. In Chinese society, cultural norms are deeply influenced by a combination of Confucian principles and modern achievement-oriented values. Core Confucian tenets, such as harmony, resilience, collectivism, and repression of personal emotions, may shape the expression of psychological distress. For instance, individuals may be socialized to internalize feelings or express them through somatic complaints rather than verbalizing emotional pain, and may be reluctant to disclose family-related adversities due to stigma and a desire to maintain family face. The environment, which is oriented towards achievement, also affects the way individuals perceive their own value. Consequently, in this specific cultural context, research on the relationship between childhood experiences and psychological well-being is of significant importance for the promotion of psychological well-being among Chinese nurses.

This study aims to identify childhood experience-based nurse subgroups and characterize their depression-burnout symptom networks to pinpoint clinical intervention targets. It addresses three research questions: (1) What are the predominant patterns of ACEs and BCEs in a sample of Chinese nurses? (2) What are the network structures of depression and burnout symptoms within each identified childhood experience subgroup? (3) How do these networks differ between subgroups in terms of core symptoms, bridge symptoms, and global network strength?

## Methods

### Participants

This cross-sectional study was conducted between September and October 2024 among nurses from a general hospital in Quanzhou City, Fujian Province, China. The study sample was selected by convenience sampling. This sampling method was employed due to practical constraints of accessibility and feasibility for this investigation. While this provides valuable preliminary insights, it may limit the generalizability of our findings to the broader population of nurses. Data for this study were obtained from the fourth wave (2024) of cross-sectional data collected during a longitudinal research project. Data from other time points in this longitudinal study have been analyzed and published in prior research [21]. The current analysis has not been previously conducted using data from this particular wave.

The following criteria were used to determine who was eligible to take part in the study: The inclusion criteria for participation in the study were as follows: (1) possession of a certificate of nursing practice in the People’s Republic of China and employment within a hospital setting; (2) no dyslexia; (3) the provision of informed consent and voluntary participation in the study. Individuals who were excluded from the study were (1) Participants with major mental illnesses were excluded to safeguard vulnerable populations per Helsinki Declaration, (2) those currently undergoing psychotherapy or taking antipsychotic medication, and (3) advanced practice nurses.

### Data collection

In this study, a total of five research instruments were employed for the purpose of data collection. All the research instruments were uploaded to the *Wenjuanxing* online platform (https://www.wjx.cn), which was specifically designed for the administration of electronic questionnaires. For submissions to be accepted, respondents were required to provide complete answers. In total, 866 valid questionnaires were obtained.

## Measurements

### Depression

The severity of depressive symptoms was evaluated using the Patient Health Questionnaire-9 item (PHQ-9) [22]. Nine items were included, each scored between 0 and 3. The scores for all items were summed to obtain the total scores. A score of 0–4 indicated the absence of depressive symptoms, 5–9 denoted mild depression, 10–14 signified moderate depression, and 15 or above indicated severe depression. The PHQ-9 has demonstrated good validity and reliability in Chinese populations [23]. In the present study, the internal consistency coefficient was 0.929.

### Burnout

Burnout levels were assessed using the Maslach Burnout Inventory (MBI), which was developed by Maslach and Jackson, and consists of three dimensions with 22 items: Emotional Exhaustion, Professional Inefficacy, and Cynicism [24]. This scale employs a seven-point rating scale. Higher scores on each dimension indicated elevated levels of burnout in the corresponding areas. The MBI scale has demonstrated good validity and reliability in Chinese populations [25]. In this study, the Cronbach’s alpha coefficients were 0.919 for the total scale, 0.949 for Emotional Exhaustion, 0.913 for professional inefficiency, and 0.895 for cynicism.

### Adverse childhood experiences

The Adverse Childhood Experiences (ACEs) scale, comprising 10 items, was employed to ascertain the presence or absence of 10 types of adverse experiences, including abuse, neglect, parental separation or divorce, and poor family functioning, occurring before the age of 18 [26]. A score of ‘0’ was assigned to the absence of experience and a score of ‘1’ was assigned to the presence of experience. The scores for the ten items on the scale were cumulatively summed to yield the individual’s total ACEs score. Higher total scores indicated more adverse experiences. The ACEs scale has demonstrated good validity and reliability in Chinese populations [27]. The internal consistency coefficient in this study was 0.942.

### Benevolent childhood experiences

The Benevolent Childhood Experience (BCEs) scale developed by Narayan et al. was used [16]. There are 10 items that assess internal (e.g., positive self-image, positive core beliefs) and family (e.g., safe caregivers, predictable family routines) resources and experiences, as well as positive relationships with friends, teachers, neighbors, relatives, or tutors. Each item is scored as 0 or 1. The scores for the ten items were summed to obtain a total BCEs score, with higher total scores indicating more positive experiences. The BCEs scale has demonstrated good validity and reliability in Chinese populations [28]. In the present study, the coefficient of consistency was 0.891.

### Control variables

Sociodemographic variables, including sex, age, marital status, education, and place of residence, were also collected.

### Statistical analysis

Descriptive analyses were used to describe the sociodemographic and psychological characteristics. Correlational analyses were used to explore correlations between continuous variables, and an Analysis of Variance (ANOVA) was used to compare differences in scores on the PHQ-9 and the three dimensions of the MBI between the demographic variables. All analyses were performed using SPSS 27.0.

LCA was conducted using Mplus 8.3, in which 20 items from the ACEs and BCEs scales were used as exogenous variables to identify potential subgroups with different characteristics of childhood experiences. The analyses began with a one-class model and iteratively added more classes to determine the most appropriate model. The smaller the values of the Akaike information criterion (AIC), Bayesian Information Criterion (BIC), and adjusted Bayesian Information Criterion (aBIC), the better is the fit. The entropy index was used to assess the classification accuracy, and its value was in the range of 0–1; the closer it was to 1, the more accurate the classification. Lo–Mendell–Rubin (LMR) and Bootstrapped Likelihood Ratio Test (BLRT) are two statistics used to compare the difference in fit between k-1 and k-category models; significant LMR and BLRT P-values indicate that the k-category model outperforms the k-1 category model, while insignificant values indicate that the k-category model does not significantly improve the fit over the k-1 category model. To ensure robustness, solutions with any latent class comprising < 10% of the sample were excluded [29].

Network analyses in R 4.3.2 examined depression-burnout symptom interactions after controlling for sociodemographic (sex/age/marital status/education/residence). Partial correlation networks visualized relationships where variables are represented by nodes, and the bivariate partial correlations between these nodes are called edges. Thicker edges indicate stronger relationships, solid/dashed edges denote positive/negative relationships. Key symptoms were identified through: (1) Centrality—measuring a symptom’s influence within its own cluster (depression or burnout), with core symptoms defined as those have higher Expected Influence (EI), which is the sum of the value of all edges connecting to one node; (2) Bridge Centrality—quantifying cross-cluster connections between depression and burnout, with bridge symptoms defined as those Bridge Expected Influence (BEI) ≥ 1 standard deviation above the mean influence. BEI indicates a node’s sum connectivity with other disorders. The network structures of depression and burnout were compared across different childhood experience subgroups through NetworkComparisonTest. The test level was set at α = 0.05. Technical procedures (e.g., regularization, stability testing) are detailed in Supplementary Methodological Appendix.

### Ethical considerations

This study adhered to all ethical guidelines and was approved by the Ethics Committee of Quanzhou First Hospital (No. 2020253). All participants were required to read and confirm their agreement to the informed consent form before accessing the online questionnaire. Participants may withdraw from the study at any time without penalty. The study used an anonymous survey model with IP address deduplication technology to prevent duplicate submissions and automatic screening of low-quality data based on completion time. The data collected was stored in an encrypted format and could only be accessed by project team members.

## Results

### Characteristics of sample

Table 1 shows general information about the nurses. The mean age of the 866 nurses was 33.65±7.83 years, 94.1% (*n* = 815) were female, 67.3% (*n* = 583) were ‘married/cohabiting’, 68.4% (*n* = 592) lived in urban areas, and 47.2% (*n* = 409) were educated to bachelor’s degree or above. Table 2 shows results of ANOVA for Clinical Scores Across Demographic Groups. ANOVA showed that those who were married/cohabiting had significantly lower levels of Professional Inefficacy than those who were single/divorced/widowed (*F* = 4.010, *P* < 0.05), those who lived in urban areas were significantly less depressed than those who lived in rural areas (*F* = 3.571, *P* < 0.05), those who lived in town had significantly higher levels of Professional Inefficacy than those who lived in rural areas (*F* = 4.871, *P* < 0.01), and those with a college education and below had significantly lower levels of Emotional Exhaustion (*F* = 6.323, *P* < 0.05) and higher levels of Professional Inefficacy (*F* = 23.233, *P* < 0.01)than those with a bachelor’s degree and above.

**Table 1:** Demographic characteristics of the sample (*N* = 866)

Items		*n*(%)/ M(SD)
Age		33.65(7.83)
Sex	Male	51(5.9)
	Female	815(94.1)
Marital Status	Married/Cohabiting	583(67.3)
	Single/Divorced/Widowed	283(32.7)
Residence	Urban	592(68.4)
	Town	149(17.2)
	Rural	125(14.4)
Education	College and below	457(52.8)
	Bachelor and above	409(47.2)

**Table 2:** Analysis of variance of clinical scores across demographic groups (*N* = 866)

Items	PHQ		Emotional exhaustion		Professional inefficacy		Cynicism	
	M(SD)	F	M(SD)	F	M(SD)	F	M(SD)	F
Sex		1.94		1.54		2.44		0.10
Male	7.78(7.03)		12.90(12.07)		30.16(11.90)		5.49(6.03)	
Female	6.60(5.80)		14.73(10.09)		27.85(10.11)		5.26(5.00)	
Marital Status		0.39		0.12		4.01*		0.84
Married/Cohabiting	6.58(5.80)		14.71(10.17)		27.50(10.41)		5.38(5.14)	
Single/Divorced/Widowed	6.85(6.07)		14.45(10.35)		28.99(9.79)		5.04(4.92)	
Residence		3.57*		1.74		4.87**		2.68
Urban	6.33(5.77)		14.19(10.28)		27.27(10.63)		5.06(4.96)	
Town	7.16(6.15)		15.30(10.16)		29.91(9.49)		5.32(4.96)	
Rural	7.73(5.99)		15.85(9.94)		29.10(8.71)		6.21(5.61)	
Education		0.37		6.32*		23.23**		2.92
College and below	6.79(6.11)		13.80(10.21)		29.55(10.34)		4.99(5.04)	
Bachelor and above	6.54(5.63)		15.54(10.17)		26.24(9.82)		5.58(5.08)	

Note. **P<0.01, *P<0.05

### Correlation analysis

Table 3 presents the results of the descriptive statistics and correlation analyses for each psychological variable. The results showed that the nurses in this study had a depression score of 6.67±5.88, an Emotional Exhaustion score of 14.62±10.22, a Professional Inefficacy score of 27. 99±10.23, a Cynicism score of 5.27±5.07, a total ACEs score of 0.60±1.89, and a total BCEs score of 8.52 ± 2.50. Depression was significantly and positively correlated with Emotional Exhaustion, Cynicism, and total ACEs scores (*P*s < 0. 01), and significantly and negatively correlated with BCEs total scores; Emotional Exhaustion was significantly and negatively correlated with Professional Inefficacy and BCEs total scores, and significantly and positively correlated with Cynicism and ACEs total scores. Professional Inefficacy was significantly and negatively correlated with cynicism, and significantly and positively correlated with total ACE scores. Cynicism was significantly negatively correlated with the total BCEs scores and positively correlated with the total ACEs scores, while the total BCEs scores were significantly negatively correlated with the total ACEs scores.

**Table 3:** Descriptive statistics and bivariate correlations among study variables (*N* = 866)

Items	M(SD)	1	2	3	4	5
1. Depression	6.67(5.88)	1				
2. Emotional Exhaustion	14.62(10.22)	0.738**	1			
3. Professional Inefficacy	27.99(10.23)	− 0.061	− 0.185**	1		
4. Cynicism	5.27(5.07)	0.633**	0.786**	− 0.100**	1	
5. BCEs total score	8.52(2.50)	− 0.399**	− 0.387**	− 0.024	− 0.345**	1
6. ACEs total score	0.60(1.89)	0.167**	0.149**	0.080*	0.157**	− 0.133**

Note. **P<0.01, *P<0.05

### LCA of childhood experiences

The fit indices for the one- to five-class models are presented in Table 4. This study selected the two-class model as optimal based on comprehensive consideration of fit statistics and model robustness. Statistical tests confirmed significant improvement over the one-class model (LMR *P* = 0.0000; BLRT *P* = 0.0000), with substantially lower information criteria values (AIC = 8146.914, BIC = 8342.233, aBIC = 8212.027). The model demonstrated good classification accuracy (entropy = 0.930) and exhibited reasonable class probability distribution (74.8%/25.2%) without extreme small classes (< 10%). While models with three or more classes showed superior fit indices, they presented potential overfitting risks: the three-class solution contained a negligible subgroup (2.7%), and both four- and five-class models yielded multiple substantively insignificant subgroups with proportions below 10% (e.g., 3.1% and 2.5% subgroups in the five-class solution). The two-class solution was ultimately adopted as it strikes an optimal balance between statistical optimization, parsimony, result stability, and theoretical interpretability.

**Fig. 1:**
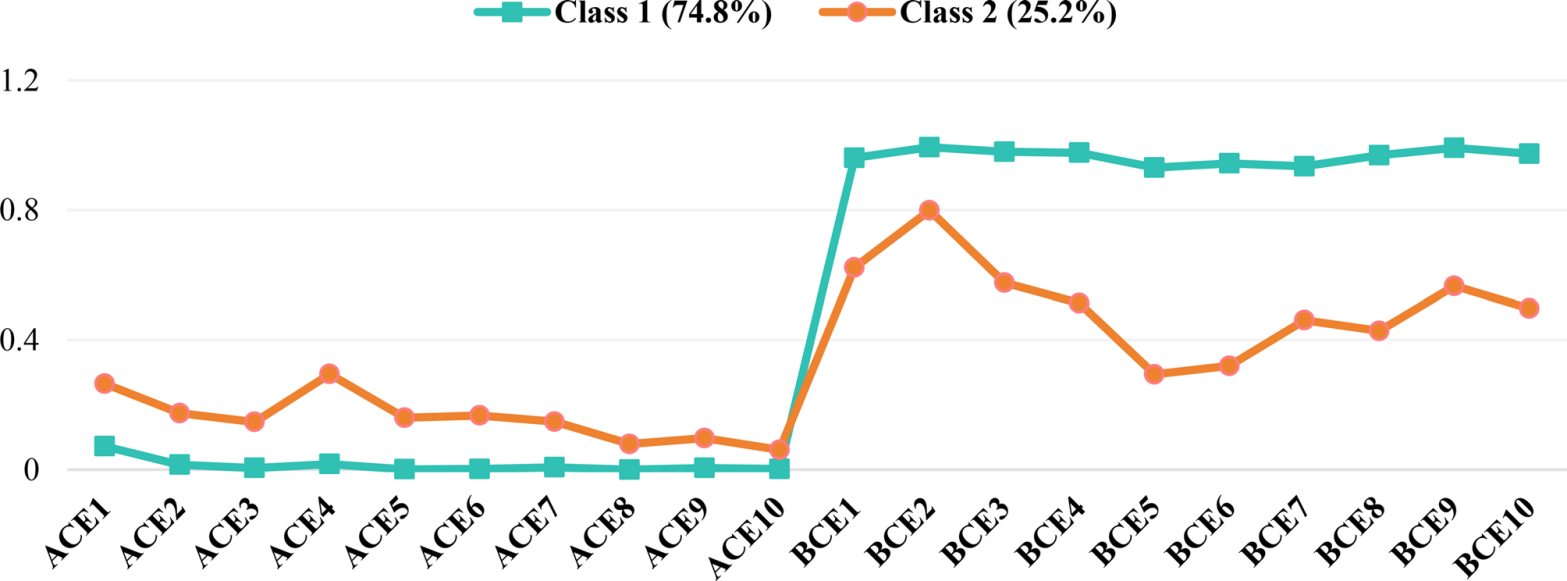
Item-response probabilities for the two-class latent model of childhood experiences

Nurses of Class 1 scored lower on all ten types of ACEs (mean range of 0.00 to 0.07) and higher on all ten items of the BCEs (mean range of 0.94 to 0.99), and were therefore classified as *Low ACEs/High BCEs*. Compared to Class 1, nurses of Class 2 scored higher on the ten ACEs types (mean range of 0.06 to 0.29), indicating a moderate level. However, their scores on the BCEs scale were lower (mean range of 0.28 to 0.81), thus being classified as *Moderate ACEs/Low BCEs*. The response patterns of the latent classes are shown in Fig. 1. Most importantly, the classification reveals two distinct, mirror-image profiles: one characterized by a highly protective childhood environment with minimal adversity, and the other defined by a relative lack of benevolent experiences coupled with modest levels of adversity. This clear dichotomy not only validates our latent class model but also underscores the co-occurring nature of adversity and the absence of benevolence in a subset of nurses, which may have important implications for their mental health outcomes.

To test the validity of the LCA in classifying the classes, a one-way ANOVA was conducted with the childhood experience subtype as the independent variable and scores on the BCEs and ACEs scales as dependent variables (see Table 5). The results showed that the main effect of the childhood experience subtype on both the BCEs and ACEs scales was significant. This suggests that there is heterogeneity within nurses’ childhood experience types, and that the two-class model is effective in distinguishing differences in childhood experience types among nurses.

An ANOVA was used to test for significant differences in depression, Emotional Exhaustion, Professional Inefficacy, and Cynicism between the two classes. Overall, individuals with *Low ACEs/High BCEs* scored significantly lower on all four scores than those with *Moderate ACEs/Low BCEs* (*P*s < 0.05).

**Table 4:** Model fit indices for latent class models from one to five classes (*N* = 866)

*N* classes	AIC	BIC	aBIC	LMR(*P*)	BLRT(*P*)	Entropy	Potential Class Probability
**1**	10348.479	10443.757	10380.242	-	-	-	1
**2**	**8146.914**	**8342.233**	**8212.027**	**0.0000**	**0.0000**	**0.930**	**0.748/0.252**
**3**	7434.374	7729.735	7532.839	0.0000	0.0000	0.964	0.027/0.763/0.210
**4**	7205.477	7600.880	7337.293	0.0000	0.0000	0.929	0.706/0.194/0.074/0.027
**5**	7081.034	7576.478	7246.200	0.0004	0.0000	0.940	0.714/0.031/0.025/0.180/0.050

**Table 5:** Comparisons of childhood experiences between the identified latent classes (*N* = 866)

Items	C1(Low ACEs/High BCEs)	C2(Moderate ACEs/Low BCEs)	F
	*n* = 648	*n* = 218	
ACEs	0.15 ± 0.47	1.95 ± 3.34	176.743^**^
BCEs	9.68 ± 0.60	5.09 ± 2.81	1521.677^**^
PHQ	5.46 ± 5.07	10.28 ± 6.62	125.219**
Emotional exhaustion	12.49 ± 8.48	20.95 ± 12.18	128.032**
Professional inefficacy	27.58 ± 10.34	29.20 ± 9.81	4.082*
Cynicism	4.34 ± 4.17	8.02 ± 6.35	95.522**

Note. **P<0.01, *P<0.05

## Network analysis of depression and burnout

### Network structure of the overall sample

**Fig. 2:**
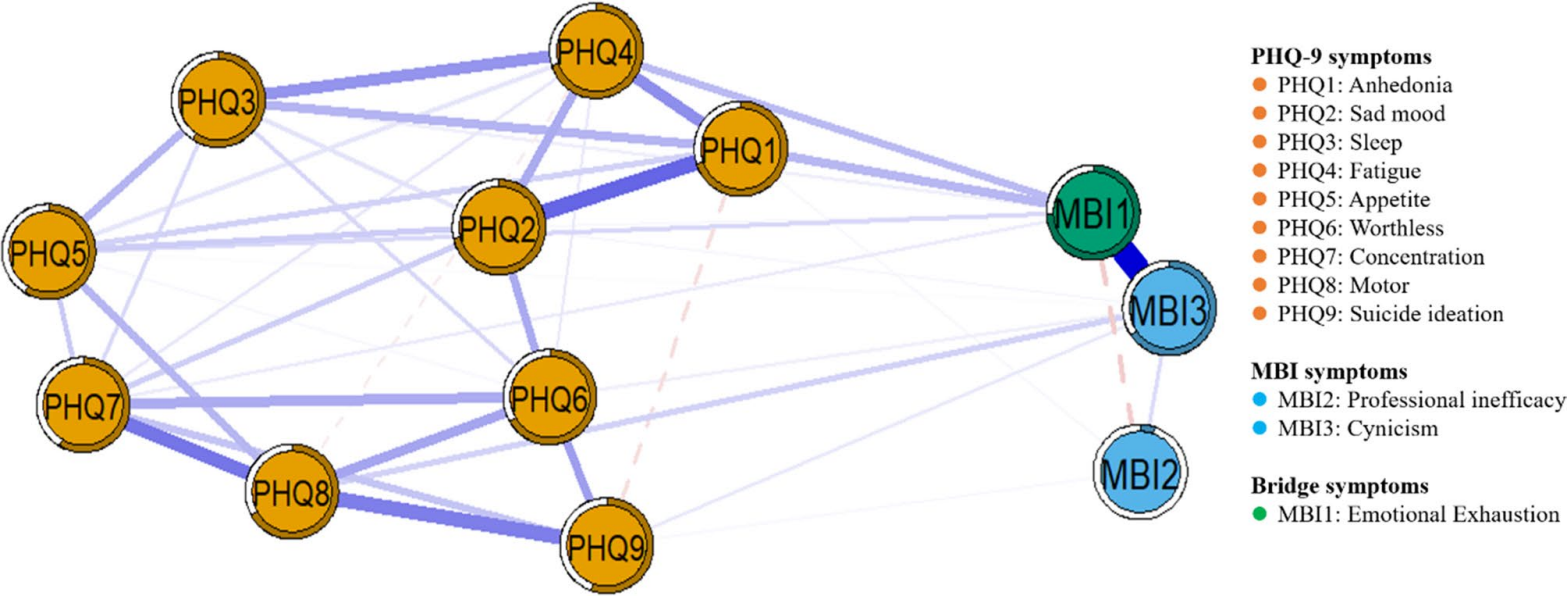
Network structure of depression and burnout symptoms in the total sample (*N* = 866). Notes. The network was estimated using the Gaussian graphical model. Nodes refer to various psychological variables. Edges refer to the bivariate partial correlations between variables. Blue lines indicate positive correlations; Red lines indicate negative correlations. Thicker edges indicate stronger correlations. PHQ, burnout, and bridge symptoms are represented by brown, blue, and green nodes, respectively. The rings around the nodes indicate the predictability of the symptom

Figure 2 shows the network structure of depression and burnout in the overall sample. Networks consist of nodes and edges. Nodes refer to various psychological variables. Edges refer to the bivariate partial correlations between variables. Of the 66 possible correlations between variables), 41 (62.12%) were significant, with 39 positive and 2 negative correlations. Between depressive symptoms and burnout symptoms, the highest correlation was observed between PHQ4 “Fatigue” and MBI1 “Emotional Exhaustion” (*r* = 0.165, see Supplementary Table 1), followed by PHQ9 “Suicide ideation” and MBI3 “Cynicism” (*r* = 0.117). Among the internal symptoms of depression, the correlation between PHQ1 “Anhedonia” and PHQ4 “Fatigue” is the strongest (*r* = 0.329), followed by PHQ8 “Psychomotor” and PHQ9 “Suicide ideation” (*r* = 0.313). Among the three dimensions of burnout, the strongest correlation is between MBI1 “Emotional Exhaustion” and MBI3 “Cynicism” (*r* = 0.560). The detailed weighted neighborhood matrix is shown in Supplementary Table 1.

### Central symptoms of the overall sample

**Fig. 3:**
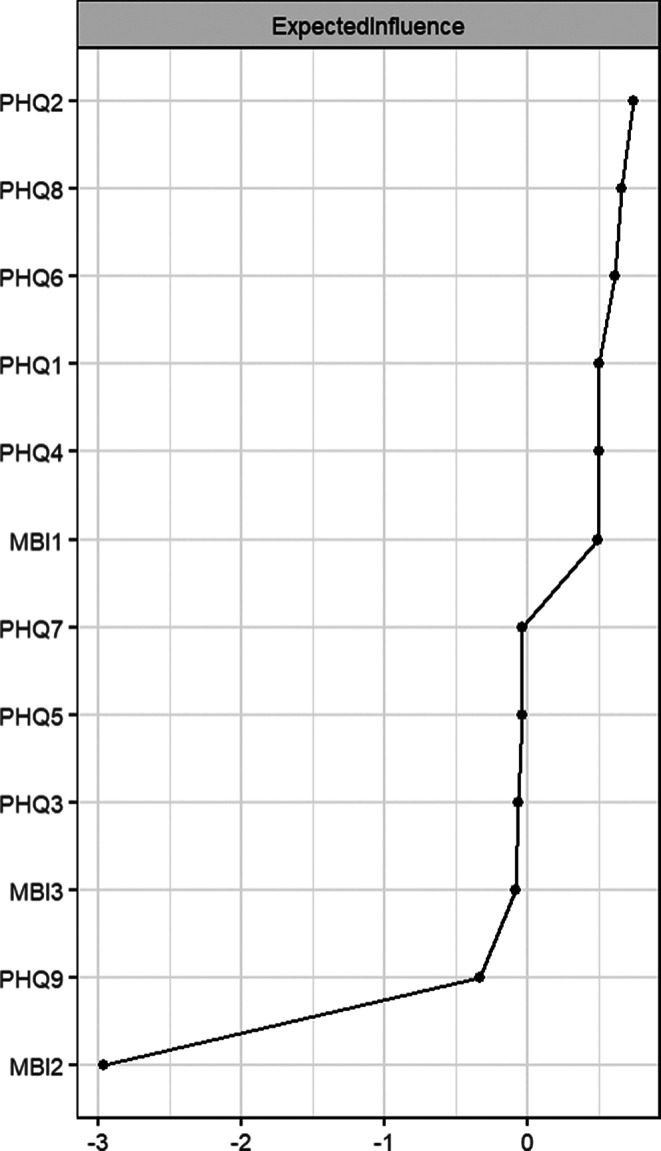
Expected influence (EI) centrality plot for the depression-burnout network in the total sample. Note. EI is expressed in *Z*-scores. Higher values indicate symptoms with a greater overall influence on the network

Nodal centrality analyses were used to assess the relative importance of nodes in a network. In the network of depression symptoms, as PHQ2 “Sad Mood” had the strongest Expected Influence (EI), “Sad Mood” emerged as central symptom of depression, which means its activity can spread and activate throughout the symptom network; in the network of burnout symptoms, MBI1 “Emotional Exhaustion” emerged as central symptom of burnout (strongest EI) (Fig. 3). Stability testing for EI indicated good stability of the overall depression-burnout network (Supplementary Fig. 1).

### Network structure of the two subgroups

**Fig. 4:**
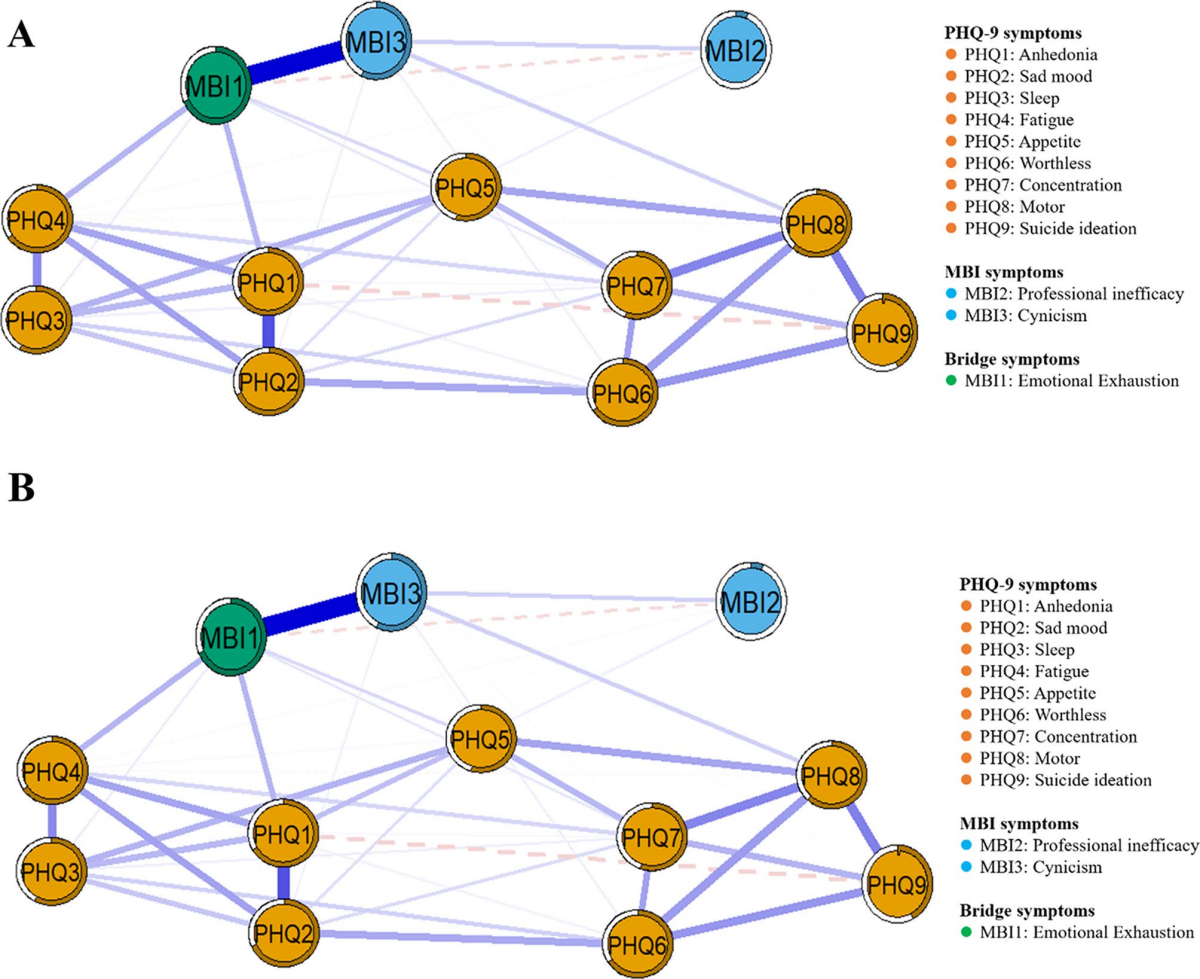
Network structures of depression and burnout symptoms across childhood experience subgroups. (**A**) Low ACEs/High BCEs group, (**B**) Moderate ACEs/Low BCEs group. Notes. See Fig. 2 legend for a detailed description of the network features

Figure 4 shows the network structure of depression and burnout in *Low ACEs/High BCEs* and *Moderate ACEs/Low BCEs* nurses. Of the 66 possible correlation, 43 (65.15%) were significant in the *Low ACEs/High BCEs* network, with 42 positive and one negative correlation, whereas 41 (62.12%) were significant in the *Moderate ACEs/Low BCEs* network, with 35 positive and six negative correlations. In the *Low ACEs/High BCEs* network for nurses, between depressive symptoms and burnout symptoms, the strongest correlation was observed between PHQ4 “Fatigue” and MBI1 “Emotional Exhaustion” (*r* = 0.155, see Supplementary Table 2), followed by PHQ7 “Concentration” and MBI1 “Emotional Exhaustion” (*r* = 0.150). Among the internal symptoms of depression, the correlation between PHQ1 “Anhedonia” and PHQ2 “Sad Mood” is the strongest (*r* = 0.346), followed by PHQ6 “Worthless” and PHQ8 “Psychomotor” (*r* = 0. 299). Among the three dimensions of burnout, the strongest correlation is between MBI1 “Emotional Exhaustion” and MBI3 “Cynicism” (*r* = 0.535). The detailed weighted neighborhood matrices are shown in Supplementary Table 2.

In the *Moderate ACEs/Low BCEs* network (see Fig. 4B), between depressive symptoms and burnout symptoms, the strongest correlation was observed between PHQ4 “Fatigue” and MBI1 “Emotional Exhaustion,” (*r* = 0.161, see Supplementary Table 3), followed by PHQ8 “Psychomotor” and MBI3 “Cynicism” (*r* = 0.147). Among the internal symptoms of depression, the correlation between PHQ1 “Anhedonia” and PHQ4 “Fatigue” is the strongest (*r* = 0. 472), followed by PHQ8 “Psychomotor” and PHQ9 “Suicide ideation” (*r* = 0. 406). Among the three dimensions of burnout, the strongest correlation is between MBI1 “Emotional Exhaustion” and MBI3 “Cynicism” (*r* = 0.527). The detailed weighted adjacency matrix is shown in Supplementary Table 3.

### Central and bridge symptoms of the two subgroups

**Fig. 5:**
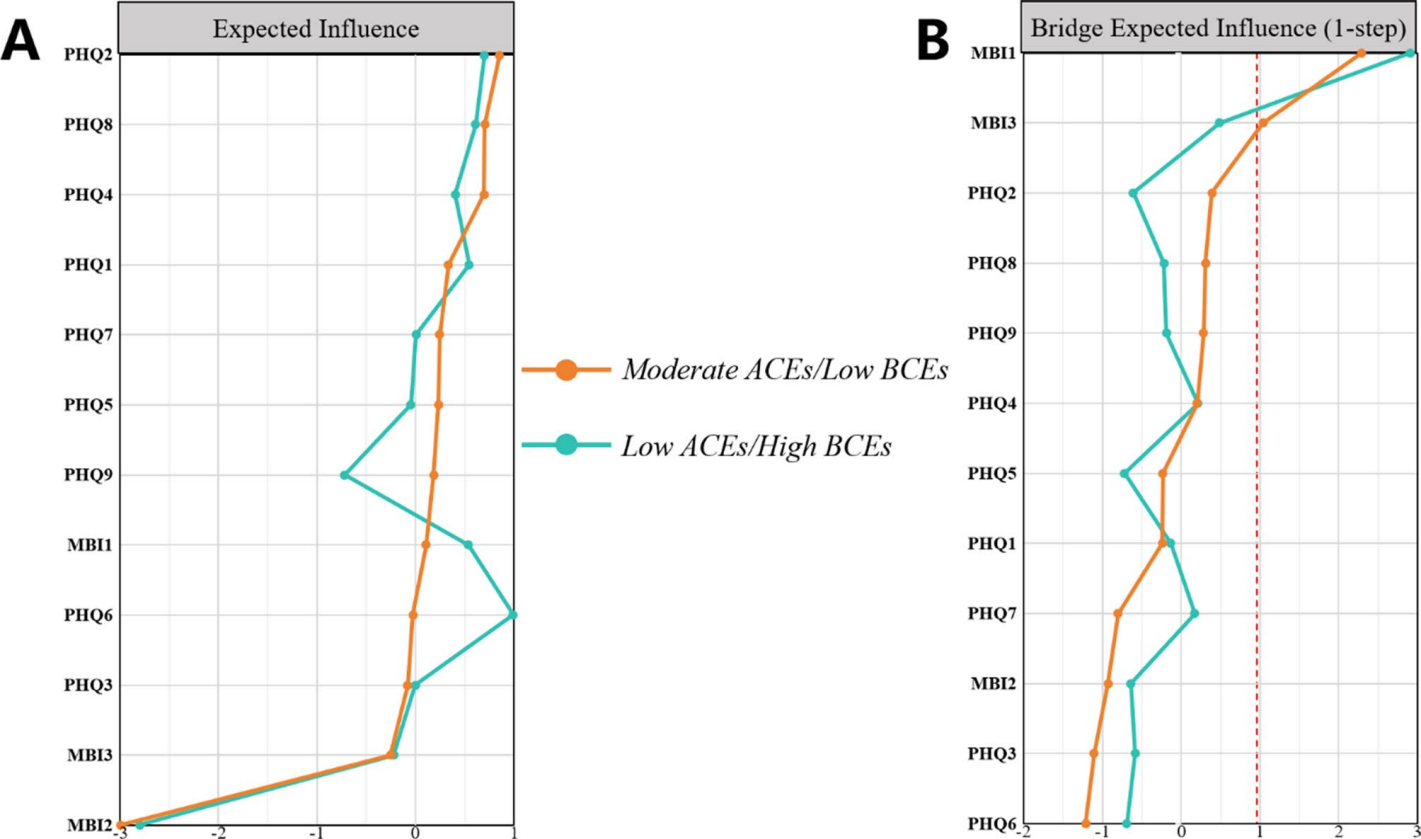
Centrality indices for the two subgroup networks. Notes. A, Expected Influence (EI) centrality. B, Bridge Expected Influence (BEI) centrality. Values are expressed in *Z*-scores. Higher values indicate symptoms with a greater overall (EI) or bridging (BEI) influence on the network. Screened bridge symptoms on the criterion of BEI (*Z*-score) ≥ 1

For the *Low ACEs/High BCEs* group, nodal centrality analyses showed that in the depression network, PHQ6 “Worthless” emerged as central symptom of depression (strongest EI), and in the burnout network, MBI1 “Emotional Exhaustion” emerged as central symptom of burnout (strongest EI) (Fig. 5A). For the *Moderate ACEs/Low BCEs* group, in the depression network, PHQ2 “Sad Mood” emerged as central symptom of depression (strongest EI); in the burnout network, MBI1 “Emotional Exhaustion” emerged as central symptom of burnout (strongest EI) (Fig. 5A). The bridging symptom centrality analysis is shown in Fig. 5B, where in the *Low ACEs/High BCEs* network only MBI1 “Emotional Exhaustion” had a BEI (*Z*-score) > 1. This indicates that the symptoms of burnout are mainly associated with the various symptoms of depression through “Emotional Exhaustion.” The *Moderate ACEs/Low BCEs* network had two symptoms with a BEI (*Z*-score) > 1: MBI1 “Emotional Exhaustion” and MBI3 “Cynicism.” This indicates that the symptoms of burnout are mainly associated with the various symptoms of depression through “Emotional Exhaustion” and “Cynicism.” Stability testing for EI and BEI indicated the depression-burnout network showed strong stability in both subgroups (Supplementary Fig. 2).

### Network comparison between the two subgroups’ network

The NetworkComparisonTest was used to assess differences in the depression-burnout network between the two subgroups. The network invariance test showed there was no significant difference between the groups (*P* = 0.22), whereas the global strength invariance test indicated that there was a significant difference between the groups (*Low ACEs/High BCEs*: 5.14 vs. *Moderate ACEs/Low BCEs*: 5.88; *S* = 0.745, *P* = 0.009). This indicates that there is a closer association between depression and burnout symptoms in the *Moderate ACEs/Low BCEs* group. The centrality invariance test (see Supplementary Table 4) showed that the EI of PHQ6 “Worthless” was significantly higher in the *Low ACEs/High BCEs* group than in the *Moderate ACEs/Low BCEs* group (*P* < 0.01), and the EI of PHQ9 “Suicide Ideation” and MBI2 “Professional Ineffectiveness” were significantly lower in *Low ACEs/High BCEs* group than in *Moderate ACEs/Low BCEs* group (*P* < 0.05).

The edge invariance test (see Supplementary Table 5) showed a significant difference between the two networks (*P* < 0.001), with a total of 11 distinct edges (correlations), five within PHQ-9 and one within MBI, and five for the correlations connecting PHQ-9 and MBI: PHQ7 “Concentration”-MBI1 “Emotional Exhaustion,” PHQ2 “Sad Mood”-MBI2 “Professional Ineffectiveness,” PHQ6 “Worthless”-MBI2 “Professional Ineffectiveness,” PHQ3 “Sleep”-MBI3 “Cynicism,” and PHQ5 “Appetite”-MBI3 “Cynicism.” The stronger correlation in the *Low ACEs/High BCEs* network were PHQ7-MBI1 and the stronger correlation in the *Moderate ACEs/Low BCEs* network were PHQ2-MBI2, PHQ6-MBI2, PHQ3-MBI3, and PHQ5-MBI3.

## Discussion

### Main findings

Our study utilized network analysis to delineate the intricate relationships between depressive and burnout symptoms among nurses with distinct childhood experiences. The key findings are threefold. First, we identified a heterogeneous but constrained distribution of childhood experiences within our sample: the vast majority of nurses (74.8%) were classified into a “*Low ACEs/High BCEs*” group, while the remainder (25.2%) fell into a “*Moderate ACEs/Low BCEs*” group. Notably, unlike comparable Western studies, our cohort did not contain a High ACEs group. Second, despite these differing backgrounds, both subgroups shared a common core of severe symptoms, most prominently MBI1 “Emotional Exhaustion”. Third, beyond this commonality, each subgroup exhibited a unique symptom network structure, with specific central and bridging symptoms, and the *Moderate ACEs/Low BCEs* group demonstrated a significantly greater overall connectivity (global strength) within and between the depression and burnout networks.

### Childhood experience profiles

LCA based on childhood experiences showed that the majority of nurses had *Low ACEs/High BCEs*, whereas a small proportion had *Moderate ACEs/low BCE*. A study by Cain also found that the dichotomous classification had the highest level of agreement in clustering criteria; specifically, 66.9% of the sample had *Low ACEs/High BCEs* and 33.1% had *High ACEs/Low BCEs* [20]. Several studies have confirmed that childhood adversity and benevolent childhood experiences are two distinct but related constructs, with higher levels of benevolent childhood experiences associated with lower levels of adversity [30, 31]. LCA analyses of ACEs and BCEs have also confirmed that the most common category in the general population is *Low ACEs/high BCEs* [19, 20]. Therefore, the data-driven approach to classification in this study effectively captured the underlying structure of childhood experiences within our sample of nurses.

### Core and consistent symptom associations across networks

Several robust and consistent connections emerged across the networks, pointing to potentially universal pathways between depression and burnout. Firstly, this study found that among the relationships between the symptoms of depression and burnout in the two groups, the partial correlation between PHQ4 “Fatigue” and MBI1 “Emotional Exhaustion” was the strongest. This association suggests that clinical nurses often experience a state of “physical and mental exhaustion”: when nurses experience persistent physical fatigue, it is often accompanied by emotional resource depletion. Several other studies have reported similar findings [32, 33]. Fatigue and emotional exhaustion are two overlapping symptoms involving energy depletion and emotional exhaustion that lead to impaired functioning. These symptoms reflect the “common endpoint” when an individual’s physical and mental resources are overwhelmed by persistent internal or external stressors [34]. Furthermore, when individuals experience fatigue or emotional exhaustion, other symptoms are more likely to emerge or intensify. Therefore, “fatigue” and “emotional exhaustion” are core symptoms that act as a key link between depression and burnout, as well as “drive” or “reinforce” other symptoms within their respective clusters. This finding underscores the importance of focusing on the core dimension of energy depletion when addressing these two common mental health issues.

Secondly, this study found that within the depressive symptom cluster, PHQ1 “Anhedonia” and PHQ2 “Sad mood,” exhibit a stable strong association, and PHQ2 “Sad mood” consistently serves as the core symptom (high EI). This pattern is consistently present in both subgroups. This result is consistent with the results of numerous studies [34–36]. Anhedonia and sad mood, as characteristic symptoms of depression, often co-occur, and their strong association may reflect a shared neurobiological basis [37]. Among these, sad mood is not only a core symptom of depression but may also serve as a pivotal node triggering or exacerbating other depressive symptoms [38].

Thirdly, among the burnout factors, MBI1 “Emotional Exhaustion” and MBI3 “Cynicism” are most strongly correlated, which has also been confirmed in previous studies [39]. This correlation indicates a strong link between the two variables. It has been suggested that cynicism is a form of expression of an individual in a state of emotional exhaustion [40], and that the presence of cynicism increases emotional exhaustion [34]. Furthermore, the MBI1 “emotional exhaustion” consistently serves as the core symptom (highest EI) in the overall network, regardless of the subgroup. This is consistent with the findings of a previous study of Chinese nurses [39]. This suggests that nodes play an important role in the activation and maintenance of the current network. The level of emotional exhaustion may affect levels of cynicism and fatigue, which may then be transmitted to other nodes, thereby affecting the entire network.

### Divergent symptom networks shaped by childhood experiences

A critical contribution of our analysis is the elucidation of how childhood experiences may shape distinct psychopathological networks. Firstly, for the *Low ACEs/High BCEs* group, PHQ6 “Worthless” is a core symptom within the depression cluster. This finding is consistent with several studies conducted in general populations [40, 41]. This finding can be interpreted through the lens of Beck’s cognitive theory of depression [42]. The theory posits that negative self-schemas and a negative cognitive triad (encompassing views of oneself, the world, and the future) are central to the development and maintenance of depression. The core role of “Worthless” in this subgroup strongly aligns with this model, suggesting that a cognitive pathway characterized by low self-evaluation is a primary driver of depressive affect in this relatively resilient group. However, it is important to note a discrepant finding: A study on a Canadian national sample identified “Sad Mood” (PHQ2) as the central symptom in the depression-anxiety network for individuals both with and without a history of childhood maltreatment [43]. This divergence highlights that the core symptomatic expression of psychological distress may vary across populations and cultural contexts, underscoring the need for further investigation into the factors underlying these differences.

Secondly, PHQ9 “Suicidal ideation” has a stronger propagative influence (high EI) in the depression network of the *Moderate ACEs/Low BCEs* group. Specifically, in this group’s network, suicidal ideation is more easily activated by other symptoms, and once it occurs, it intensifies other symptoms more strongly. Adverse childhood experiences impair an individual’s emotional regulation abilities, leading to entrenched negative cognitions and a loss of hope, coupled with a lack of positive resources to buffer these effects, making suicidal ideation more easily triggered and influencing the overall network. From a neurobiological perspective, the *Moderate ACEs/Low BCEs* group may exhibit hypothalamic-pituitary-adrenal (HPA) axis dysfunction, prefrontal cortex functional inhibition, and increased default mode network activity, along with heightened rumination [44–46]. These changes make suicidal ideation more easily triggered and harder to suppress.

Thirdly, the propagative influence (high EI) of MBI2 “Professional Inefficacy” and MBI3 “Cynicism” were significantly stronger in the *Moderate ACEs/Low BCEs* network than in the *Low ACEs/High BCEs* network. Research has shown that adversity increases an individual’s sense of worthlessness [47] and reduces empathy (reflected in cynicism [48]), and this increased variability has led to the association of MBI2 “Professional Inefficacy” or MBI3 “Cynicism” with depression factors increased. According to Domke, adversity predicts levels of rumination in adulthood, and rumination is a factor that strengthens the link between an individual’s “Professional Inefficacy” and “Sad Mood” or “Worthlessness” [49]. Individuals with higher levels of adversity showed an increased effect of MBI3 “Cynicism” on depression, which is consistent with the findings of a study on nursing students [50]. Cynicism reduces an individual’s tolerance to the stress of potential events, increases sensitivity to negative events, and intensifies stress responses and the production of somatic responses such as sleep and appetite abnormalities. Childhood adversity intensifies the stress response, making cynicism a strong predictor of depression-related symptoms [51]. The results of this study suggest that, in addition to focusing on emotional exhaustion in nurses with high levels of adversity, two other dimensions of burnout, Cynicism and Professional Inefficacy, should also receive more attention.

Fourthly, the global strength invariance test indicated that individuals in *Moderate ACEs/Low BCEs* group had more entanglement both within and between depression and burnout. Similar results have been reported in previous studies. For example, a study of a national Canadian sample found that a group with a history of childhood maltreatment had stronger connections between the depression and anxiety networks [43]. Xue et al. found that anxiety and depressive symptoms were more strongly linked in older adults in the high comorbidity group than in those in the low depression-anxiety comorbidity group [52]. According to the job demands-resources theory, a mismatch between job demands and coping resources may lead to burnout and negative emotions [53]. Adverse childhood experiences can reduce an individual’s resources, trapping them in a cycle of depletion and widening the gap between job demands and resources, thereby strengthening the link between burnout and depression [54]. Additionally, childhood experiences can influence an individual’s cognitive and emotional regulation. Prolonged traumatic experiences can weaken an individual’s emotional regulation, causing emotions related to depression and stress responses related to burnout to interact and reinforce each other. A lack of protective resources during childhood can cause individuals to develop negative cognitive characteristics. They may generalize localized, limited stress events into comprehensive, uncontrollable existential crises. This further blurs the pathological boundaries between depression and burnout. This makes it challenging to develop effective preventive interventions for depression and burnout.

### The role of culture on distress expression and experience

Cultural factors offer a compelling framework for understanding the distinctive distribution of childhood experiences and the structure of symptom networks in our sample. The population of nurses in this study reported lower ACEs scores and therefore did not have a High ACEs group as found in the two western studies mentioned above. The lower ACEs scores were also consistent with the findings of several Chinese studies [55, 56]. The distribution pattern of childhood experiences revealed in this study essentially reflects the combined influence of characteristics of the nursing profession and Chinese cultural adaptation. The absence of a high ACEs group may not indicate its true nonexistence. We hypothesize that it could instead suggest dual filtering through self-selection bias and cultural under-reporting. On the one hand, individuals with high ACEs may self-select out of high-pressure helping professions, such as nursing, or they may exhibit higher attrition rates due to the long-term physiological and psychological tolls. On the other hand, collectivist cultural norms foster tendencies to minimize trauma, leading to systematically lower ACEs scale scores. Future research with targeted recruitment strategies and culturally sensitive measures is needed to definitively explore this potential dual filtering effect.

The observed divergence in core symptoms—where PHQ6 “Worthless” was central in our sample versus PHQ2 “Sad Mood” in a Western counterpart—also can be understood through a cultural lens. In China’s collectivist society, cultural values place a greater emphasis on the role and obligations of the individual within the family and social group. Within this framework, failing to meet collective expectations or fulfill one’s duties can profoundly impact self-worth. Therefore, the prominence of “Worthless” as a core symptom in our cohort may be a culturally salient manifestation of distress. It potentially reflects a internalized sense of having failed in one’s social roles, which then activates and is reinforced by other depressive symptoms such as sadness, fatigue, and even suicidal ideation. In contrast, the centrality of “Sad Mood” in individualistic Western societies might reflect a more direct and inwardly-focused expression of emotional suffering. This cross-cultural variation underscores the importance of interpreting network findings within their specific socio-cultural context.

### Implications

The findings of this study carry significant implications for clinical practice and theoretical understanding.

Our results enable precise, subgroup-specific mental health interventions for nurses. We propose a multi-tiered framework: Primary prevention for all staff involves integrating routine brief monitoring of emotional exhaustion (e.g., using the MBI-EE subscale or BAT screener) and fatigue management strategies (e.g., enforced breaks, workload balancing tools) into workflows. Secondary prevention targeting at-risk staff requires systematic screening for childhood trauma history, followed by prioritized structured assessment of core symptoms (e.g., with the PHQ-9 for depression/suicide risk) and offering targeted interventions such as brief CBT or ACT skills groups. Tertiary prevention for symptomatic staff necessitates ensuring rapid access to intensive support (e.g., Employee Assistance Program counselors, psychiatric referral) and accommodations for those with severe symptoms or crisis.

Theoretically, this study advances understanding by showing that childhood experiences (ACEs/BCEs) shape not only symptom severity but the very architecture of psychopathological networks, influencing central symptoms and cluster connections. The distinct patterns of shared (e.g., Emotional Exhaustion) and unique (e.g., Worthlessness vs. Sad Mood) core symptoms across subgroups provide robust support for person-centered models, moving beyond uniform comorbidity frameworks and highlighting developmental history as a key determinant of heterogeneous clinical presentations.

## Limitations

This study had several limitations. First, the cross-sectional design prevents longitudinal tracking of symptom networks, offering no insight into how connections (e.g., between fatigue and emotional exhaustion) evolve over time. Future longitudinal studies using panel network analyses are needed to model these dynamic processes. Second, the use of convenience sampling, while practical for initial exploration, may limit the generalizability of our findings. Future studies should employ randomized or stratified probability sampling designs to ensure demographic representativeness and enhance the external validity of results across diverse populations. Third, the ratings used in this study were self-reported and may have been subject to recall bias and social desirability. Future research could incorporate objective measures or triangulate self-reports with interviews or observational methods to mitigate subjective reporting biases. Fourth, the sample sizes of the two groups divided by LCA differed significantly (648 vs. 218 participants), with a smaller sample size in the *Moderate ACEs/Low BCEs* group; A larger sample size is needed to validate future results, which researchers could achieve by collaborating across multiple sites to recruit balanced cohorts.

### Strengths

This study had several strengths. To the best of our knowledge, this is the first study to combine LCA and network analysis to explore the relationship between depression and burnout among nurses with different childhood experiences. The results of this study may help us gain a deeper understanding of individual differences and focus on the specific symptoms exhibited by nurses rather than on the simple level of psychological problems reflected by the total score on a scale, thus guiding the development of more targeted and specific intervention strategies.

## Conclusions

In summary, we found similarities and uniqueness in the network structures of depression and burnout among nurses with different childhood experiences. MBI1 “Emotional Exhaustion” served as the core symptom and bridging symptom connecting depression and burnout in both network groups. The association between depression and burnout was stronger in *Moderate ACEs/Low BCEs* group than in *Low ACEs/High BCEs* group.

## Supplementary Information

Below is the link to the electronic supplementary material.

Supplementary Material 1

Supplementary Material 2

Supplementary Material 3

Supplementary Material 4

## Data Availability

The datasets generated and analysed during the current study are not publicly available due privacy and ethical restrictions of the participants, but are available from the corresponding author on reasonable request.

## Abbreviations

ACE: Adverse Childhood Experiences
ACEQ-R: Revised Adverse Childhood Experience Questionnaire
AIC: Akaike Information Criterion
ANOVA: Analysis of Variance
BCE: Benevolent Childhood Experience
BEI: Bridge Expected Influence
BIC: Bayesian Information Criterion
BLRT: Bootstrapped Likelihood Ratio Test
BMI: Body Mass Index
CS: Correlation Stability Coefficient
EI: Expected Influence
GLASSO: Graphical Least Absolute Shrinkage and Selection Operator
ICD: International Classification of Diseases
LCA: Latent Class Analysis
LMR: Lo-Mendell-Rubin
MBI: Maslach Burnout Inventory
NCT: Network Comparison Test
PHQ: Patient Health Questionnaire
