# Supplementary material for: Childhood experience profiles and their impact on depression–burnout networks among nurses: a latent class and network analysis
Source: BMC Nurs. 2025 Sep 29;24:1216. doi: 10.1186/s12912-025-03889-x (PMC12482161; doi:10.1186/s12912-025-03889-x)
Supplement: Supplementary file 1 — Supplementary Material 1 [file 12912_2025_3889_MOESM1_ESM.docx]

**Supplementary table details for further processing**

Supplementary Table 1. Weight matrix for the regularized partial correlation network estimation of the total sample (n=866)

Supplementary Table 2. Weight matrix for regularized partial correlation network estimation of *Low ACEs/High BCEs* (n=648)

Supplementary Table 3. Weight matrix for regularized partial correlation network estimation of *Moderate ACEs/Low BCEs* (n=218)

Supplementary Table 4. The centrality invariance test of ExpectedInfluence between two networks

Supplementary Table 5. The edge invariance test between two networks

**Supplementary figure details for further processing**

Supplementary Figure 1. The overall network CS for EI and BEI

Supplementary Figure 2. The network CS for EI and BEI of the two groups
