# Supplementary material for: Childhood experience profiles and their impact on depression–burnout networks among nurses: a latent class and network analysis
Source: BMC Nurs. 2025 Sep 29;24:1216. doi: 10.1186/s12912-025-03889-x (PMC12482161; doi:10.1186/s12912-025-03889-x)

Supplementary Figure 1. The overall network CS for EI and BEI


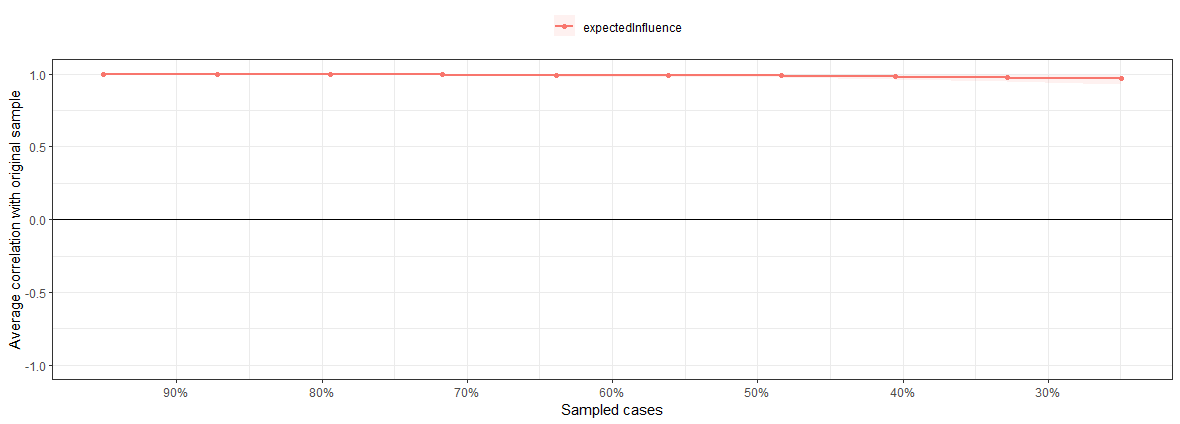


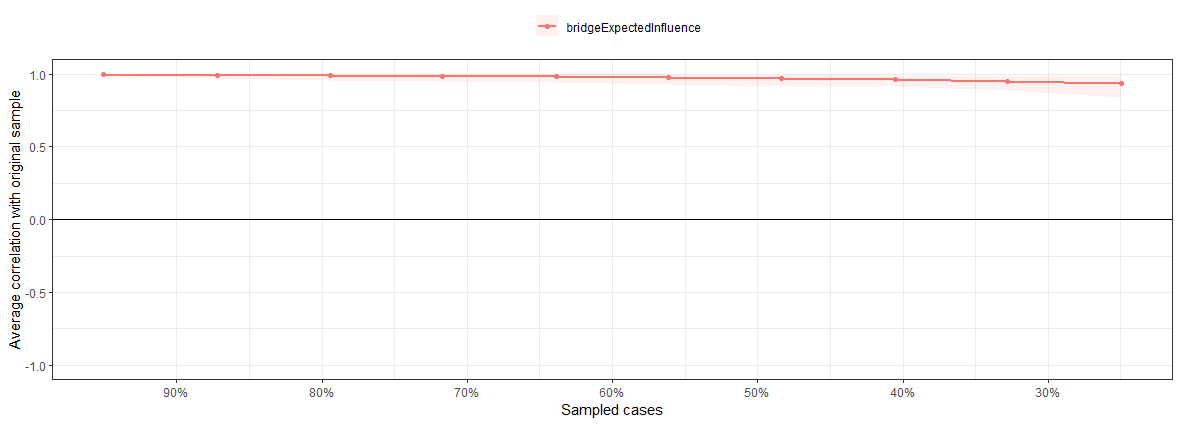


Supplementary Figure 2. The network CS for EI and BEI of the two groups


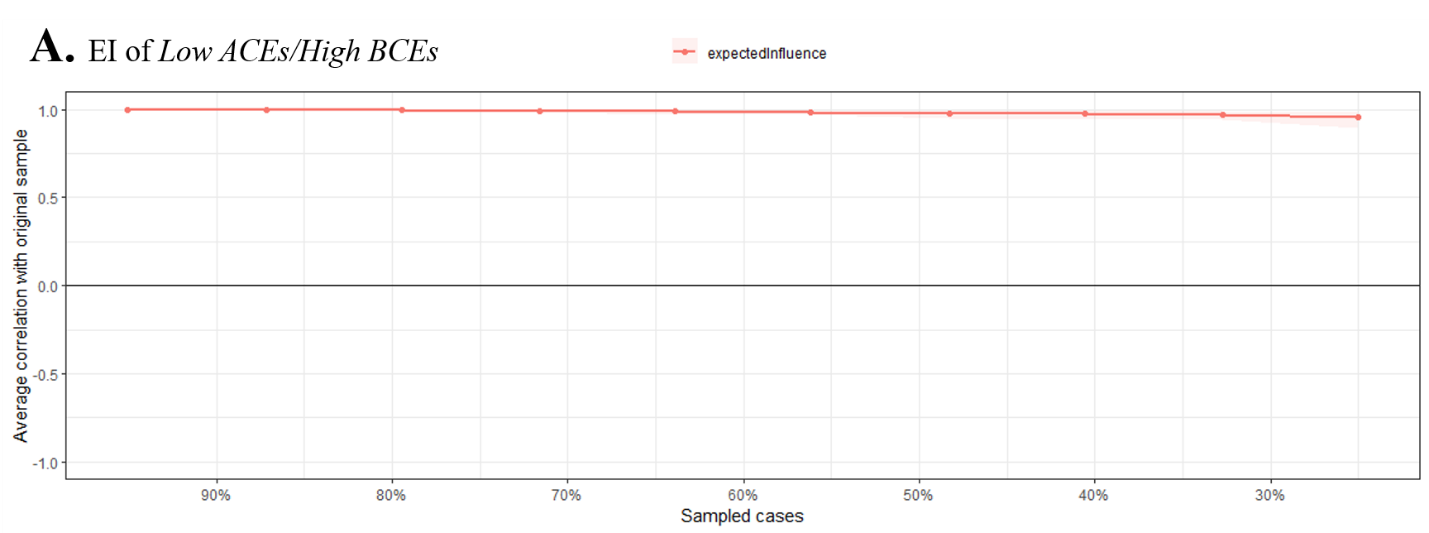


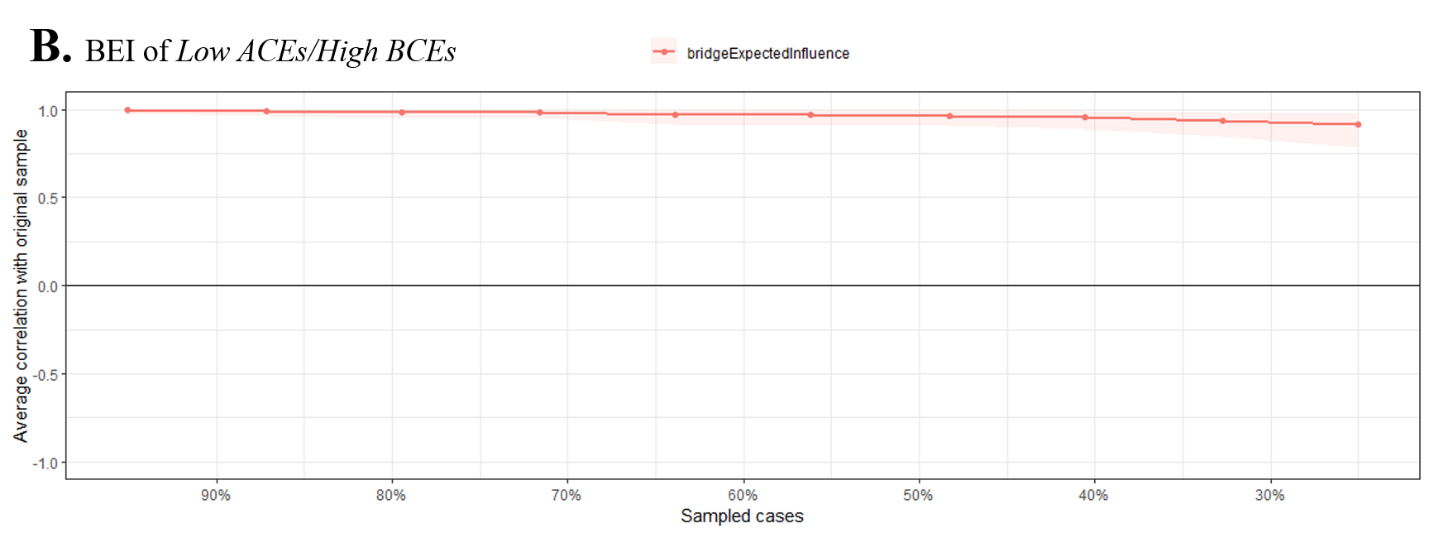

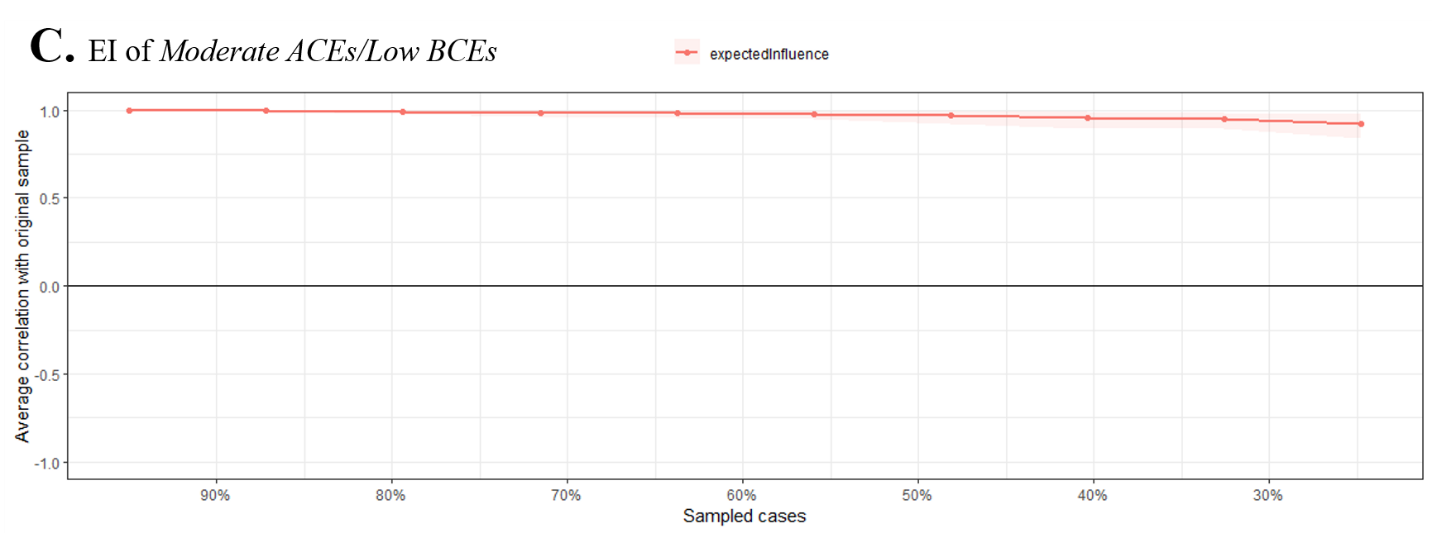


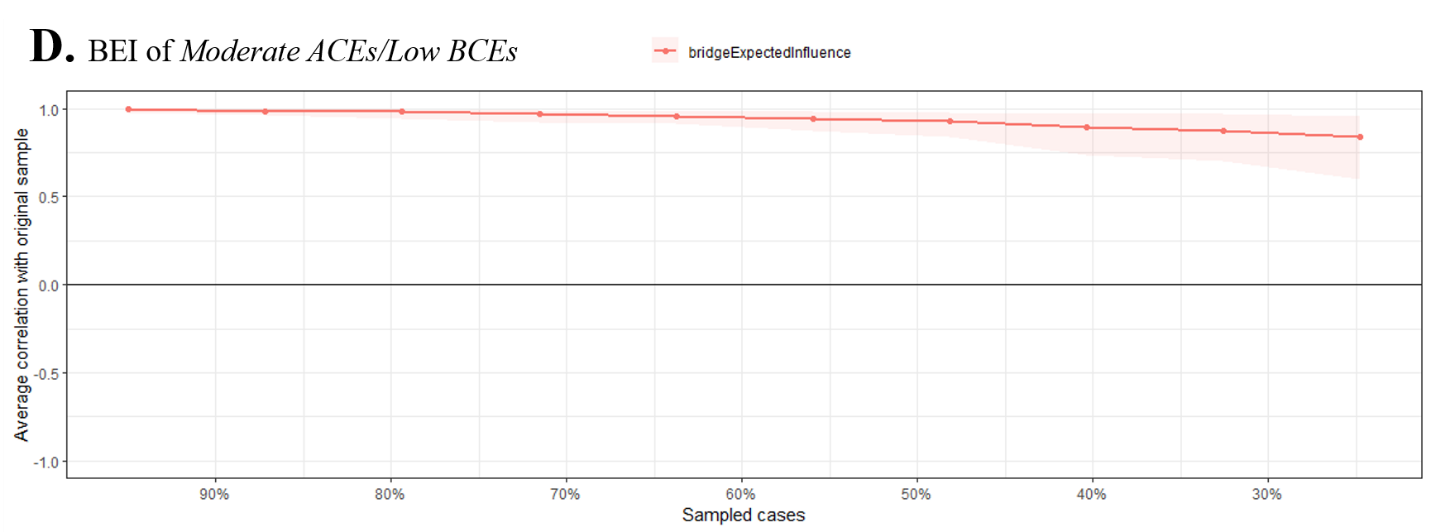

Supplement: Supplementary file 2 — Supplementary Material 2 [file 12912_2025_3889_MOESM2_ESM.docx]
