# Supplementary material for: Childhood experience profiles and their impact on depression–burnout networks among nurses: a latent class and network analysis
Source: BMC Nurs. 2025 Sep 29;24:1216. doi: 10.1186/s12912-025-03889-x (PMC12482161; doi:10.1186/s12912-025-03889-x)
Supplement: Supplementary file 3 — Supplementary Material 3 [file 12912_2025_3889_MOESM3_ESM.docx]

**Supplementary Methodological Appendix—Network Analysis**

Network analyses were performed using R 4.3.2. Network structure relationships between depression and burnout were explored using network analysis. First, the *qgraph* package was used to construct the network structure. To reduce spurious correlations, the Graphical Least Absolute Shrinkage and Selection Operator (GLASSO) was used to obtain and visualize a regularized network. Given that sociodemographic factors may influence comorbidity patterns of depression and burnout [17], we performed an adjusted network analysis after controlling for sex, age, place of residence, marital status, and education. Following Xue (2024), we regressed each of the nine items of the PHQ-9 and the three dimensions of the MBI as dependent variables, regressed the aforementioned sociodemographic variables as independent variables, converted each of the dependent variables into residuals, standardized them using *z*-transformation, and used them as nodes for network analysis [18]. The connecting lines (edges) represent partial correlations between nodes. The edges in the network can be positive (solid lines) or negative (dashed lines), and the thickness of the edges indicates the strength of the relationship between nodes. Second, the Expected Influence (EI) and Bridge Expected Influence (BEI) were calculated. EI is calculated by *qgraph*; the higher the EI, the greater the importance of the node in the network. The BEI is calculated using *networktools* and is usually presented as a *Z*-score. A higher BEI value indicates that the node has a greater activation of other network symptoms. Following the methodology of a previous study [19], a BEI *Z*-score of ≥1 represents the symptom as a bridge symptom. The *bootnet* package was used to estimate the accuracy of edge weight estimates and the stability of centrality indices, and to calculate the correlation stability coefficient (CS), which was greater than 0.25, representing an acceptable range of stability, and greater than 0.5 indicating good stability. Finally, based on the LCA classification, the *NetworkComparisonTest* (NCT) package was used to compare the network structures of depression and burnout among different subgroups of childhood experiences. The test level was set at α= 0.05.
