# Supplementary material for: Childhood experience profiles and their impact on depression–burnout networks among nurses: a latent class and network analysis
Source: BMC Nurs. 2025 Sep 29;24:1216. doi: 10.1186/s12912-025-03889-x (PMC12482161; doi:10.1186/s12912-025-03889-x)
Supplement: Supplementary file 4 — Supplementary Material 4 [file 12912_2025_3889_MOESM4_ESM.docx]

Supplementary Table 1. Weights matrix for the regularized partial correlation network estimation of total sample (n=866)

|  | **PHQ1** | **PHQ2** | **PHQ3** | **PHQ4** | **PHQ5** | **PHQ6** | **PHQ7** | **PHQ8** | **PHQ9** | **MBI1** | **MBI2** | **MBI3** |
| --- | --- | --- | --- | --- | --- | --- | --- | --- | --- | --- | --- | --- |
| **PHQ1** | 0.000 |  |  |  |  |  |  |  |  |  |  |  |
| **PHQ2** | 0.299 | 0.000 |  |  |  |  |  |  |  |  |  |  |
| **PHQ3** | 0.203 | 0.046 | 0.000 |  |  |  |  |  |  |  |  |  |
| **PHQ4** | 0.329 | 0.140 | 0.241 | 0.000 |  |  |  |  |  |  |  |  |
| **PHQ5** | 0.027 | 0.137 | 0.161 | 0.147 | 0.000 |  |  |  |  |  |  |  |
| **PHQ6** | 0.000 | 0.208 | 0.041 | 0.000 | 0.157 | 0.000 |  |  |  |  |  |  |
| **PHQ7** | 0.004 | 0.079 | 0.114 | 0.000 | 0.086 | 0.141 | 0.000 |  |  |  |  |  |
| **PHQ8** | 0.000 | 0.054 | 0.000 | -0.029 | 0.077 | 0.215 | 0.296 | 0.000 |  |  |  |  |
| **PHQ9** | 0.000 | 0.022 | 0.000 | 0.000 | 0.000 | 0.238 | 0.014 | 0.313 | 0.000 |  |  |  |
| **MBI1** | 0.102 | 0.082 | 0.013 | 0.165 | 0.036 | 0.009 | 0.096 | 0.000 | 0.033 | 0.000 |  |  |
| **MBI2** | 0.029 | 0.000 | 0.000 | 0.000 | 0.000 | 0.000 | 0.000 | 0.000 | 0.000 | -0.103 | 0.000 |  |
| **MBI3** | 0.000 | 0.000 | 0.000 | 0.000 | 0.000 | 0.021 | 0.000 | 0.116 | 0.117 | 0.560 | 0.000 | 0.000 |

Supplementary Table 2. Weights matrix for the regularized partial correlation network estimation of *Low ACEs/High BCEs* (n=648)

|  | **PHQ1** | **PHQ2** | **PHQ3** | **PHQ4** | **PHQ5** | **PHQ6** | **PHQ7** | **PHQ8** | **PHQ9** | **MBI1** | **MBI2** | **MBI3** |
| --- | --- | --- | --- | --- | --- | --- | --- | --- | --- | --- | --- | --- |
| **PHQ1** | 0.000 |  |  |  |  |  |  |  |  |  |  |  |
| **PHQ2** | 0.346 | 0.000 |  |  |  |  |  |  |  |  |  |  |
| **PHQ3** | 0.194 | 0.088 | 0.000 |  |  |  |  |  |  |  |  |  |
| **PHQ4** | 0.251 | 0.150 | 0.275 | 0.000 |  |  |  |  |  |  |  |  |
| **PHQ5** | 0.049 | 0.138 | 0.144 | 0.095 | 0.000 |  |  |  |  |  |  |  |
| **PHQ6** | 0.000 | 0.208 | 0.039 | 0.000 | 0.133 | 0.000 |  |  |  |  |  |  |
| **PHQ7** | 0.046 | 0.055 | 0.046 | 0.026 | 0.091 | 0.148 | 0.000 |  |  |  |  |  |
| **PHQ8** | 0.000 | 0.006 | 0.000 | 0.000 | 0.126 | 0.299 | 0.261 | 0.000 |  |  |  |  |
| **PHQ9** | 0.000 | 0.005 | 0.000 | 0.000 | 0.016 | 0.265 | 0.011 | 0.224 | 0.000 |  |  |  |
| **MBI1** | 0.104 | 0.043 | 0.046 | 0.155 | 0.028 | 0.000 | 0.150 | 0.000 | 0.000 | 0.000 |  |  |
| **MBI2** | 0.004 | 0.000 | 0.000 | 0.000 | 0.000 | 0.011 | 0.000 | 0.000 | 0.023 | -0.069 | 0.000 |  |
| **MBI3** | 0.000 | 0.000 | 0.000 | 0.000 | 0.000 | 0.020 | 0.000 | 0.096 | 0.077 | 0.535 | 0.040 | 0.000 |

Supplementary Table 3. Weights matrix for the regularized partial correlation network estimation of *Moderate ACEs/Low BCEs* (n=218)

|  | **PHQ1** | **PHQ2** | **PHQ3** | **PHQ4** | **PHQ5** | **PHQ6** | **PHQ7** | **PHQ8** | **PHQ9** | **MBI1** | **MBI2** | **MBI3** |
| --- | --- | --- | --- | --- | --- | --- | --- | --- | --- | --- | --- | --- |
| **PHQ1** | 0.000 |  |  |  |  |  |  |  |  |  |  |  |
| **PHQ2** | 0.190 | 0.000 |  |  |  |  |  |  |  |  |  |  |
| **PHQ3** | 0.193 | 0.000 | 0.000 |  |  |  |  |  |  |  |  |  |
| **PHQ4** | 0.472 | 0.132 | 0.143 | 0.000 |  |  |  |  |  |  |  |  |
| **PHQ5** | 0.000 | 0.134 | 0.206 | 0.231 | 0.000 |  |  |  |  |  |  |  |
| **PHQ6** | -0.031 | 0.220 | 0.056 | 0.000 | 0.163 | 0.000 |  |  |  |  |  |  |
| **PHQ7** | 0.000 | 0.095 | 0.211 | 0.000 | 0.052 | 0.138 | 0.000 |  |  |  |  |  |
| **PHQ8** | 0.000 | 0.132 | 0.000 | -0.086 | 0.000 | 0.099 | 0.326 | 0.000 |  |  |  |  |
| **PHQ9** | 0.000 | 0.018 | 0.000 | 0.000 | 0.000 | 0.199 | 0.061 | 0.406 | 0.000 |  |  |  |
| **MBI1** | 0.090 | 0.137 | 0.010 | 0.161 | 0.000 | 0.006 | 0.000 | 0.031 | 0.061 | 0.000 |  |  |
| **MBI2** | 0.000 | 0.054 | 0.000 | 0.000 | 0.000 | -0.074 | 0.000 | 0.000 | 0.000 | -0.194 | 0.000 |  |
| **MBI3** | 0.000 | 0.000 | -0.059 | 0.000 | 0.091 | 0.003 | 0.000 | 0.147 | 0.113 | 0.527 | -0.130 | 0.000 |

Supplementary Table 4. The centrality invariance test of ExpectedInfluence between two networks

|  | **EI（*Low ACEs/High BCEs*）** | **EI（*Moderate ACEs/Low BCEs*）** | **P*-*value** |
| --- | --- | --- | --- |
| **PHQ1** | 0.994 | 0.915 | 0.456 |
| **PHQ2** | 1.039 | 1.113 | 0.457 |
| **PHQ3** | 0.832 | 0.759 | 0.408 |
| **PHQ4** | 0.952 | 1.052 | 0.355 |
| **PHQ5** | 0.820 | 0.877 | 0.475 |
| **PHQ6** | 1.124 | 0.779 | 0.006 |
| **PHQ7** | 0.835 | 0.882 | 0.702 |
| **PHQ8** | 1.013 | 1.055 | 0.699 |
| **PHQ9** | 0.621 | 0.859 | 0.018 |
| **MBI1** | 0.992 | 0.829 | 0.109 |
| **MBI2** | 0.009 | -0.344 | 0.001 |
| **MBI3** | 0.768 | 0.693 | 0.470 |

Supplementary Table 5. The edge invariance test between two networks

| **Var1** | **Var2** | **Edge_weights**  **（*Low ACEs/High BCEs*）** | **Edge_weights**  **（*Moderate ACEs/Low BCEs*）** | **P-value** |
| --- | --- | --- | --- | --- |
| PHQ1 | PHQ2 | 0.346 | 0.190 | 0.085 |
| PHQ1 | PHQ3 | 0.194 | 0.193 | 0.993 |
| PHQ1 | PHQ4 | 0.251 | 0.472 | 0.030 |
| PHQ1 | PHQ5 | 0.049 | 0.000 | 0.463 |
| PHQ1 | PHQ6 | 0.000 | -0.031 | 0.030 |
| PHQ1 | PHQ7 | 0.046 | 0.000 | 0.178 |
| PHQ1 | PHQ8 | 0.000 | 0.000 | 1.000 |
| PHQ1 | PHQ9 | 0.000 | 0.000 | 1.000 |
| PHQ1 | MBI1 | 0.104 | 0.090 | 0.863 |
| PHQ1 | MBI2 | 0.004 | 0.000 | 0.950 |
| PHQ1 | MBI3 | 0.000 | 0.000 | 1.000 |
| PHQ2 | PHQ3 | 0.088 | 0.000 | 0.221 |
| PHQ2 | PHQ4 | 0.150 | 0.132 | 0.817 |
| PHQ2 | PHQ5 | 0.138 | 0.134 | 0.969 |
| PHQ2 | PHQ6 | 0.208 | 0.220 | 0.880 |
| PHQ2 | PHQ7 | 0.055 | 0.095 | 0.630 |
| PHQ2 | PHQ8 | 0.006 | 0.132 | 0.054 |
| PHQ2 | PHQ9 | 0.005 | 0.018 | 0.875 |
| PHQ2 | MBI1 | 0.043 | 0.137 | 0.174 |
| PHQ2 | MBI2 | 0.000 | 0.054 | 0.019 |
| PHQ2 | MBI3 | 0.000 | 0.000 | 1.000 |
| PHQ3 | PHQ4 | 0.275 | 0.143 | 0.150 |
| PHQ3 | PHQ5 | 0.144 | 0.206 | 0.467 |
| PHQ3 | PHQ6 | 0.039 | 0.056 | 0.823 |
| PHQ3 | PHQ7 | 0.046 | 0.211 | 0.007 |
| PHQ3 | PHQ8 | 0.000 | 0.000 | 1.000 |
| PHQ3 | PHQ9 | 0.000 | 0.000 | 1.000 |
| PHQ3 | MBI1 | 0.046 | 0.010 | 0.380 |
| PHQ3 | MBI2 | 0.000 | 0.000 | 1.000 |
| PHQ3 | MBI3 | 0.000 | -0.059 | 0.011 |
| PHQ4 | PHQ5 | 0.095 | 0.231 | 0.115 |
| PHQ4 | PHQ6 | 0.000 | 0.000 | 1.000 |
| PHQ4 | PHQ7 | 0.026 | 0.000 | 0.320 |
| PHQ4 | PHQ8 | 0.000 | -0.086 | 0.043 |
| PHQ4 | PHQ9 | 0.000 | 0.000 | 1.000 |
| PHQ4 | MBI1 | 0.155 | 0.161 | 0.940 |
| PHQ4 | MBI2 | 0.000 | 0.000 | 1.000 |
| PHQ4 | MBI3 | 0.000 | 0.000 | 1.000 |
| PHQ5 | PHQ6 | 0.133 | 0.163 | 0.736 |
| PHQ5 | PHQ7 | 0.091 | 0.052 | 0.691 |
| PHQ5 | PHQ8 | 0.126 | 0.000 | 0.080 |
| PHQ5 | PHQ9 | 0.016 | 0.000 | 0.459 |
| PHQ5 | MBI1 | 0.028 | 0.000 | 0.661 |
| PHQ5 | MBI2 | 0.000 | 0.000 | 1.000 |
| PHQ5 | MBI3 | 0.000 | 0.091 | 0.033 |
| PHQ6 | PHQ7 | 0.148 | 0.138 | 0.915 |
| PHQ6 | PHQ8 | 0.299 | 0.099 | 0.041 |
| PHQ6 | PHQ9 | 0.265 | 0.199 | 0.477 |
| PHQ6 | MBI1 | 0.000 | 0.006 | 0.884 |
| PHQ6 | MBI2 | 0.011 | -0.074 | 0.004 |
| PHQ6 | MBI3 | 0.020 | 0.003 | 0.818 |
| PHQ7 | PHQ8 | 0.261 | 0.326 | 0.528 |
| PHQ7 | PHQ9 | 0.011 | 0.061 | 0.325 |
| PHQ7 | MBI1 | 0.150 | 0.000 | 0.018 |
| PHQ7 | MBI2 | 0.000 | 0.000 | 1.000 |
| PHQ7 | MBI3 | 0.000 | 0.000 | 1.000 |
| PHQ8 | PHQ9 | 0.224 | 0.406 | 0.087 |
| PHQ8 | MBI1 | 0.000 | 0.031 | 0.189 |
| PHQ8 | MBI2 | 0.000 | 0.000 | 1.000 |
| PHQ8 | MBI3 | 0.096 | 0.147 | 0.483 |
| PHQ9 | MBI1 | 0.000 | 0.061 | 0.256 |
| PHQ9 | MBI2 | 0.023 | 0.000 | 0.173 |
| PHQ9 | MBI3 | 0.077 | 0.113 | 0.694 |
| MBI1 | MBI2 | -0.069 | -0.194 | 0.052 |
| MBI1 | MBI3 | 0.535 | 0.527 | 0.910 |
| MBI2 | MBI3 | 0.040 | -0.130 | 0.001 |
